# Cardiac fibrosis: mechanistic insights and translational advances

**DOI:** 10.1186/s43556-026-00587-1

**Published:** 2026-09-16

**Authors:** Hui-Yi Xie, Xue-Ting Zheng, Xiu-Heng Wang, Jia-Yan Yang, Yun-Xi Liu, Yue Zhao, Hui-Fang Tang, Heng-Jing Hu

**Affiliations:** 1 The First Affiliated Hospital, Department of Cardiovascular Medicine, Hengyang Medical School, University of South China, No. 69 Chuanshan Road, Shigu District, Hengyang, Hunan 421001 P.R. China; 2 The First Affiliated Hospital, Department of Endocrine, Hengyang Medical School, University of South China, Hengyang, Hunan 421001 P.R. China; 3 Department of Cardiovascular Disease and Key Laboratory for Atherosclerosis of Hunan Province, Hengyang Medical School, University of South China, Hengyang, Hunan 421001 P.R. China

**Keywords:** Cardiac fibrosis, SIRT3, Mitochondrial homeostasis, Oxidative stress, Extracellular matrix, Translational therapy

## Abstract

Cardiac fibrosis is a critical pathological process driving the progression of heart failure, characterized by excessive extracellular matrix deposition, collagen network remodeling, and expansion of the myocardial interstitium. While fibrosis may reflect a reparative response to tissue injury, it can also signify a maladaptive remodeling process that progressively impairs cardiac structure and function. Cardiac fibroblasts and myofibroblasts serve as the principal effector cells responsible for matrix accumulation, whereas cardiomyocytes and immune cells contribute to fibrotic expansion through inflammatory signaling, paracrine communication, and microenvironmental regulation. Transforming growth factor-β, the renin–angiotensin–aldosterone system, inflammatory cytokines, mechanical stress, and metabolic disturbances collectively orchestrate a profibrotic signaling network. By regulating extracellular matrix synthesis, degradation, and crosslinking, these pathways promote myocardial stiffening and functional decompensation. In this review, we discuss the histopathological patterns of cardiac fibrosis across various pathological contexts, the major cellular contributors, and the core molecular mechanisms involved, while summarizing current advances in diagnostic evaluation and translational therapeutic strategies. We further discuss the context-dependent role of sirtuin 3 (SIRT3), a mitochondrial nicotinamide adenine dinucleotide (NAD⁺)-dependent deacetylase, in linking mitochondrial homeostasis, oxidative stress, metabolic adaptation, and profibrotic signaling. More precise antifibrotic strategies require better definition of fibroblast states, fibrosis activity, and disease-specific remodeling patterns.

## Introduction

Cardiac fibrosis is characterized by expansion of the myocardial interstitium resulting from net accumulation of extracellular matrix (ECM) proteins and may reflect either reparative or maladaptive remodeling processes [1]. Its pathological characteristics vary substantially across disease contexts and stages. Despite this heterogeneity, its development is governed by extensive crosstalk among multiple profibrotic signaling networks, including the transforming growth factor-β (TGF-β)/Smad pathway, the renin–angiotensin–aldosterone system (RAAS), mechanotransduction pathways, inflammatory and immune signaling, and the axis linking oxidative stress, mitochondrial dysfunction, and metabolic reprogramming.

The convergence of these signaling pathways with diverse cellular responses makes cardiac fibrosis difficult to characterize adequately using any single biomarker or molecular pathway. Although circulating biomarkers and multiparametric imaging techniques have improved the assessment of fibrotic burden, their clinical utility remains constrained by limited cardiac specificity, insufficient standardization, and suboptimal disease stratification. These limitations also suggest that interventions directed at a single downstream pathway may be inadequate to address the multilayered and dynamically evolving pathological network underlying cardiac fibrosis. In this context, SIRT3, a major mitochondrial NAD^+^-dependent deacetylase, has received increasing attention because it regulates several processes closely associated with fibrogenesis, including redox homeostasis, metabolic adaptation, mitochondrial quality control, inflammation, and profibrotic signaling [2–5]. Nevertheless, the context-dependent functions of SIRT3 across different fibrotic conditions, its direct and indirect effects in distinct cardiac cell populations, and the challenges involved in translating experimental findings into clinical applications remain incompletely understood.

This review first outlines the major pathological contexts and histological patterns of cardiac fibrosis and then examines the principal cellular contributors and core molecular mechanisms involved in fibrotic remodeling. It subsequently focuses on the role of SIRT3 in fibrotic processes associated with mitochondrial dysfunction and metabolic remodeling. Finally, current diagnostic approaches and translational therapeutic strategies are discussed to provide a structured framework for understanding disease-specific fibrotic remodeling and informing the development of more precisely targeted antifibrotic interventions.

## Cardiac fibrosis in different pathological contexts

Cardiac fibrosis is not a uniform pathological process. Its predominant histological patterns, underlying drivers, and functional consequences differ among disease settings. Histologically, cardiac fibrosis can be broadly classified into two major forms: replacement fibrosis and interstitial fibrosis. Replacement fibrosis primarily reflects scar formation after irreversible cardiomyocyte loss, whereas interstitial fibrosis is more commonly associated with chronic pressure overload, metabolic disturbances, and age-related remodeling. The heterogeneity of cardiac fibrosis should therefore be interpreted in relation to the underlying disease and the nature of myocardial injury.

### Replacement fibrosis following Myocardial Infarction (MI)

MI causes ischemic necrosis and irreversible loss of cardiomyocytes. Because the adult mammalian myocardium has limited regenerative capacity, the necrotic region undergoes a sequential reparative response involving inflammatory clearance of cellular debris, fibroblast recruitment and activation, myofibroblast differentiation, and collagen deposition, ultimately leading to the formation of an extracellular matrix (ECM)-rich scar [6, 7]. Post-infarction fibrosis is therefore predominantly considered a form of replacement or reparative fibrosis. Scar formation is initially adaptive because it stabilizes the damaged myocardium and reduces the risk of ventricular wall rupture. However, when fibrotic remodeling extends beyond the infarcted region and persists in the border and remote myocardium, it can become maladaptive. Progressive scar expansion and ECM accumulation contribute to left ventricular dilation, reduced myocardial compliance, greater heterogeneity of electrical conduction, and ultimately the development and progression of heart failure [8].

### Interstitial and perivascular fibrosis associated with pressure overload/hypertension

Unlike the focal replacement scar that develops after MI, fibrosis associated with chronic pressure overload or hypertension typically manifests as diffuse reactive interstitial and perivascular fibrosis [9]. Hypertension, aortic stenosis, and other pressure-overload conditions expose the myocardium to persistent mechanical stretch and increased wall stress. These stimuli promote cardiomyocyte hypertrophy and interact with neurohumoral activation, inflammatory signaling, and fibroblast activation to drive progressive ECM remodeling [10, 11]. The principal pathological features include increased collagen deposition between cardiomyocytes and expansion of the perivascular matrix. Early or moderate matrix remodeling may help preserve cardiac structure, whereas persistent collagen accumulation and increased cross-linking increase ventricular stiffness and impair diastolic function [12].

### Fibrosis associated with diabetes and metabolic dysregulation

Cardiac fibrosis associated with diabetes and metabolic disorders does not necessarily begin with acute cardiomyocyte necrosis. Instead, it develops gradually in response to chronic hyperglycemia, insulin resistance, lipotoxicity, and low-grade inflammation [13, 14]. Hyperglycemia can activate cardiac fibroblasts and promote ECM deposition through several interrelated mechanisms, including the signaling mediated by advanced glycation end products (AGEs) and the receptor for AGEs (RAGE), oxidative stress, mitochondrial dysfunction, microvascular endothelial injury, RAAS activation, and increased collagen cross-linking [15]. This form of fibrosis can develop even in the absence of overt obstructive coronary artery disease or hypertension and is associated with increased myocardial stiffness, diastolic dysfunction, and progression of diabetic cardiomyopathy.

### Heart Failure with preserved Ejection Fraction (HFpEF) and age-related fibrotic remodeling

Fibrosis associated with HFpEF and aging typically does not manifest as a focal scar attributable to a single etiology. Rather, it reflects diffuse myocardial remodeling driven by multiple chronic stressors. HFpEF frequently coexists with comorbidities such as advanced age, hypertension, obesity, diabetes, and chronic kidney disease [16]. These factors can promote interstitial and perivascular matrix accumulation through systemic low-grade inflammation, coronary microvascular endothelial dysfunction, impaired NO-cGMP-PKG signaling [17], and sustained fibroblast activation [18]. The aging myocardium is additionally characterized by impaired collagen turnover, increased collagen cross-linking, and senescence-associated inflammation, all of which may contribute to increased myocardial stiffness [19]. Fibrosis associated with HFpEF and aging should therefore be viewed as a chronic and diffuse structural substrate closely linked to metabolic and vascular dysfunction.

## Cellular actors in cardiac fibrosis

Cardiac fibrosis is a continuous and dynamic process involving multiple cell populations, ECM remodeling, and regulation that varies according to disease context [1]. Among these cell types, fibroblasts and myofibroblasts are the principal effector cells responsible for ECM deposition and scar formation. Cardiomyocytes, macrophages, and other cell populations modulate fibrotic remodeling primarily through injury-related responses and regulation of the local microenvironment.

### Cardiac fibroblasts

Cardiac fibroblasts constitute one of the major non-cardiomyocyte cell populations in the adult heart. They are located predominantly within the endomysial and perimysial interstitium but are also found in the vascular adventitia, atria, annulus fibrosus, and valves [20]. Although fibroblasts represent only a proportion of the total cardiac cell population, they are essential for maintaining myocardial structure and mechanical properties. Through coordinated collagen synthesis and remodeling, they contribute to ECM homeostasis [21, 22] and help preserve normal cardiac conduction and rhythm [23]. Their homeostatic functions under physiological conditions nevertheless remain incompletely understood. Single-cell transcriptomic studies indicate that fibroblasts in the healthy heart are not a homogeneous population of quiescent cells but comprise multiple phenotypic states. Fibroblast activation during cardiac fibrosis is similarly dynamic, giving rise to distinct cellular states at different stages of scar formation [24, 25].

The functional state of cardiac fibroblasts varies considerably across disease contexts. Following MI [25, 26], early fibroblast activation is required for effective wound healing and scar stabilization. In this setting, fibroblast-derived ECM replaces tissue lost after cardiomyocyte death and helps preserve ventricular wall integrity. Persistent or excessive fibroblast activation, however, can promote fibrotic remodeling in the infarct border zone and remote myocardium. Under conditions of pressure overload or hypertension [9, 10], sustained mechanical and neurohumoral stress promotes diffuse interstitial and perivascular fibrosis rather than focal scar formation. In diabetes, aging, and HFpEF [13, 15, 16, 18, 19], chronic fibroblast activation is more closely associated with low-grade inflammation, endothelial dysfunction, oxidative stress, and metabolic dysregulation.

Cardiac fibroblasts therefore serve both as essential mediators of tissue repair and as major drivers of maladaptive fibrotic remodeling. Accordingly, the central therapeutic question is not whether fibroblast activity should be broadly suppressed, but rather which fibroblast states should be targeted, at what stage, and in which anatomical or pathological context. Future antifibrotic approaches will need to distinguish transient reparative fibroblast activation from persistent pathological activation. This distinction is particularly relevant to therapies targeting fibroblast signaling, ECM turnover, mechanotransduction, and mitochondrial or metabolic regulation.

### Myofibroblasts

In the injured heart, myofibroblasts are generally regarded as an activated fibroblast state induced by tissue injury or chronic stress rather than as a distinct cellular lineage [26]. Compared with quiescent fibroblasts, myofibroblasts exhibit greater contractile activity and substantially increased ECM-producing capacity. Lineage-tracing studies have shown that most myofibroblasts in the injured heart originate from resident fibroblasts. Studies of MI further indicate that fibroblast activation and transition toward the myofibroblast phenotype are temporally regulated and highly dynamic, with cellular phenotype and function evolving throughout the course of scar formation and repair [25]. These findings suggest that myofibroblasts should not be regarded simply as a passive terminal state induced by injury; rather, their emergence and persistence are governed by tightly regulated molecular programs. Consistent with this concept, bulk and single-cell clustered regularly interspaced short palindromic repeat (CRISPR) screens have identified several chromatin regulators that control the transition of primary cardiac fibroblasts from a quiescent state to a fibrotic phenotype. These findings indicate that acquisition of the myofibroblast state depends not only on extracellular cytokine stimulation but also on coordinated transcriptional and epigenetic regulation [27]. Further studies indicate that the epigenetic regulator TET3 promotes fibroblast-to-myofibroblast transition through transcriptional activation of the mechanosensitive ion channel Piezo2, thereby enhancing cardiac fibrosis. This finding supports a mechanistic link between mechanotransduction and epigenetic regulation during myofibroblast differentiation [28]. Taken together, myofibroblasts are better understood as a dynamic and potentially reversible cellular state rather than simply as terminal effector cells. Selective regulation of pathological myofibroblast states may therefore offer new therapeutic opportunities.

### Other cellular contributors

Although fibroblasts and myofibroblasts are the principal matrix-producing cells in the fibrotic heart, their behavior is strongly influenced by signals derived from surrounding cardiomyocytes, immune cells, endothelial cells, and vascular-associated cell populations. These cells may not themselves produce substantial amounts of ECM, but they shape the fibrotic microenvironment through the release of cytokines, chemokines, growth factors, extracellular vesicles, and injury-associated molecular signals. Such multicellular regulation is particularly important because fibroblast activation often arises from integrated tissue-level stress responses rather than from exposure to a single molecular stimulus. Recent studies indicate that cardiomyocytes function not only as targets of injury but also as active paracrine regulators of fibrotic remodeling. Senesi et al. reported that cardiomyocyte-derived extracellular vesicles containing miR-24-3p can be taken up by cardiac fibroblasts and may regulate fibroblast activation. Ischemia-like stimulation reduced miR-24-3p levels in these vesicles and weakened this protective effect [29]. Conversely, necrotic or severely injured cardiomyocytes release damage-associated molecular patterns (DAMPs), which can activate cardiac fibroblasts and promote local inflammatory and fibrotic responses [30, 31].

Compared with cardiomyocytes, macrophages contribute to cardiac fibrosis primarily by remodeling the post-injury microenvironment. Recent reviews have emphasized that macrophage-fibroblast interactions are involved throughout cardiac homeostasis, injury repair, and adverse remodeling and are characterized by substantial heterogeneity, plasticity, and temporal dependence [32, 33]. Early studies suggested that, during MI repair, a subset of macrophages may acquire fibroblast-like features and express collagen- and fibrosis-associated markers, raising the possibility that macrophages might contribute directly to scar formation [34]. More recent evidence, however, favors the interpretation that these cells acquire a profibrotic phenotype rather than undergo true conversion into fibroblasts. Using lineage tracing and single-cell analysis, Li et al. demonstrated that macrophages in the infarcted heart can acquire fibrosis-associated transcriptional programs and express multiple matricellular proteins without substantial fibroblast conversion [35]. Consistent with these findings, Hoeft et al. identified a population of secreted phosphoprotein 1-positive (SPP1⁺) macrophages with pronounced profibrotic properties in a cross-organ fibrosis model. Cxcl4 deficiency reduced the emergence of these profibrotic macrophages and attenuated fibrosis following cardiac and renal injury [36]. Collectively, these observations indicate that macrophages contribute to fibrosis mainly by adopting profibrotic states and modulating fibroblast activation rather than by directly becoming major matrix-producing cells.

Endothelial and microvascular alterations also contribute substantially to fibrotic remodeling [18, 37]. Coronary microvascular endothelial dysfunction can reduce nitric oxide bioavailability, impair NO-cGMP-PKG signaling [17], and create a local environment that promotes inflammation, oxidative stress, cardiomyocyte stiffness, and fibroblast activation. This mechanism is particularly relevant to HFpEF and metabolic heart disease, in which fibrosis is typically diffuse and closely associated with systemic comorbidities rather than focal cardiomyocyte necrosis. Perivascular fibrosis may likewise result from sustained crosstalk among endothelial cells, vascular smooth muscle cells, inflammatory cells, and adventitial fibroblasts [38]. Vascular dysfunction can therefore act both as an initiating stimulus and as an amplifier of interstitial and perivascular matrix remodeling.

This multicellular perspective has important therapeutic implications. Direct inhibition of fibroblasts alone may be insufficient when upstream cardiomyocyte injury, immune activation, endothelial dysfunction, or metabolic stress remains unresolved. Conversely, interventions that attenuate oxidative stress, inflammation, neurohumoral activation, or mitochondrial dysfunction may indirectly suppress fibroblast activation by improving the local tissue microenvironment. These interactions may partly explain why antifibrotic strategies exert different effects across cardiac diseases and pathological contexts.

## Molecular mechanisms driving cardiac fibrosis

**Fig. 1:**
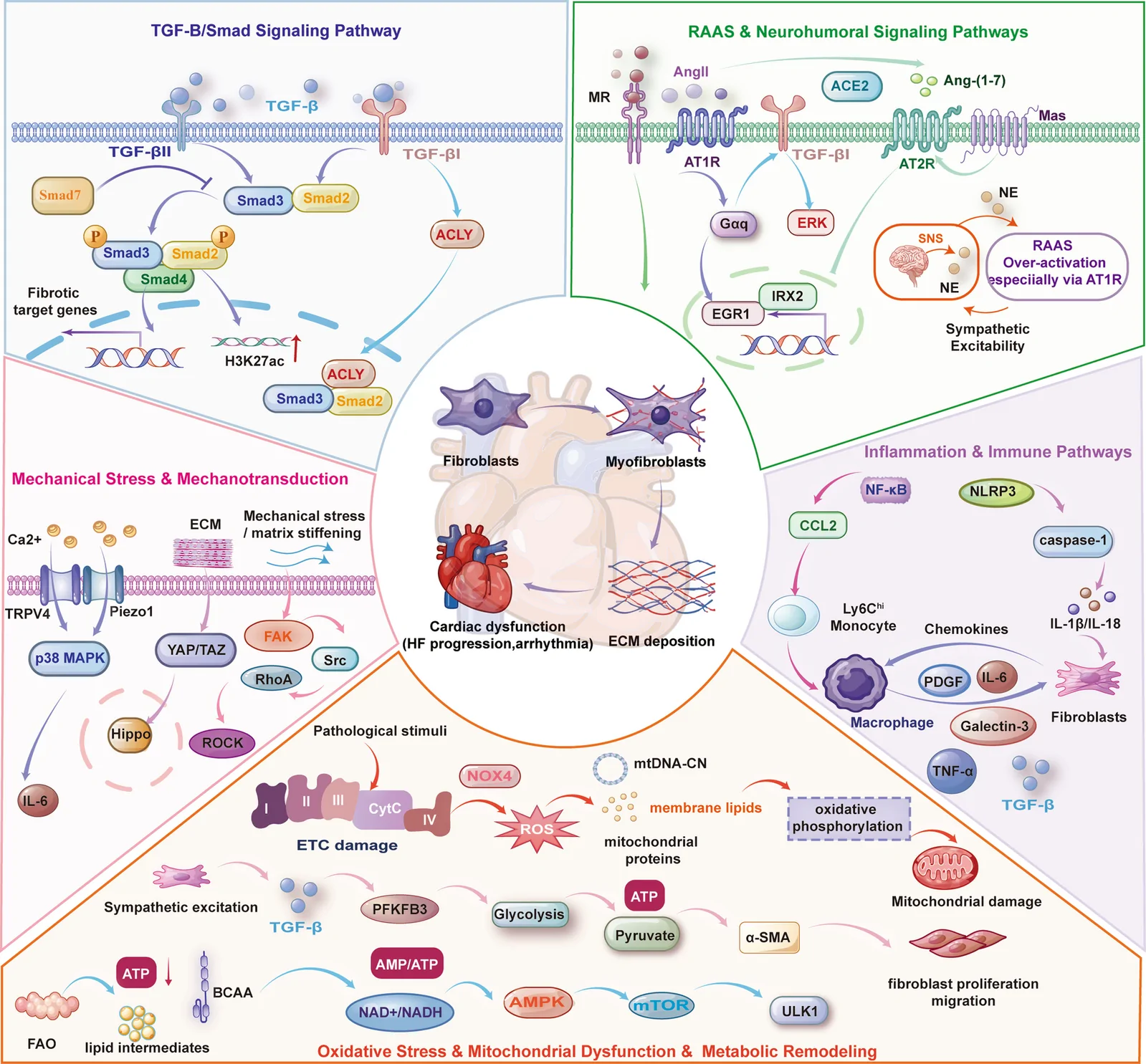
Major molecular mechanisms driving cardiac fibrosis. Schematic overview of the major pathways involved in cardiac fibrosis. The figure summarizes transforming growth factor-β (TGF-β)/Smad signaling, renin–angiotensin–aldosterone system (RAAS) and neurohumoral signaling, mechanical stress and mechanotransduction, inflammatory and immune pathways, and oxidative stress, mitochondrial dysfunction, and metabolic remodeling, which collectively contribute to fibroblast activation, myofibroblast transition, extracellular matrix (ECM) deposition, and cardiac dysfunction. Abbreviations: ACE2, angiotensin-converting enzyme 2; ACLY, ATP citrate lyase; AMPK, AMP-activated protein kinase; AMP, adenosine monophosphate; Ang II, angiotensin II; Ang-(1–7), angiotensin-(1–7); AT1R, angiotensin II type 1 receptor; AT2R, angiotensin II type 2 receptor; ATP, adenosine triphosphate; BCAA, branched-chain amino acid; CCL2, C–C motif chemokine ligand 2; CytC, cytochrome c; ECM, extracellular matrix; EGR1, early growth response 1; ERK, extracellular signal-regulated kinase; ETC, electron transport chain; FAK, focal adhesion kinase; FAO, fatty acid oxidation; Gαq, G protein alpha q subunit; H3K27ac, histone H3 lysine 27 acetylation; IL-1β, interleukin-1β; IL-6, interleukin-6; IL-18, interleukin-18; IRX2, iroquois homeobox 2; MAPK, mitogen-activated protein kinase; MR, mineralocorticoid receptor; mtDNA-CN, mitochondrial DNA copy number; mTOR, mechanistic target of rapamycin; NAD⁺, nicotinamide adenine dinucleotide; NADH, reduced nicotinamide adenine dinucleotide; NE, norepinephrine; NF-κB, nuclear factor-κB; NLRP3, NLR family pyrin domain containing 3; NOX4, NADPH oxidase 4; PDGF, platelet-derived growth factor; PFKFB3, 6-phosphofructo-2-kinase/fructose-2,6-bisphosphatase 3; RAAS, renin–angiotensin–aldosterone system; RhoA, Ras homolog family member A; ROCK, Rho-associated coiled-coil-containing protein kinase; ROS, reactive oxygen species; SNS, sympathetic nervous system; TAZ, transcriptional coactivator with PDZ-binding motif; TGF-β, transforming growth factor-β; TNF-α, tumor necrosis factor-α; TRPV4, transient receptor potential vanilloid 4; ULK1, UNC-51-like kinase 1; YAP, Yes-associated protein; α-SMA, alpha-smooth muscle actin

Cardiac fibrosis can arise from a wide range of pathological stimuli, but its initiation and progression are sustained by coordinated changes in multiple profibrotic pathways. TGF-β/Smad signaling, the RAAS and other neurohumoral pathways, mechanotransduction, and inflammatory and immune responses interact to regulate fibroblast activation and ECM remodeling. Increasing evidence also links these processes to disturbances in redox homeostasis, mitochondrial function, and cellular metabolism, which can in turn modify profibrotic signaling and fibroblast behavior. Together, these mechanisms form an interconnected regulatory network governing the development and persistence of cardiac fibrosis (Fig. 1).

### TGF-β/Smad signaling

The TGF-β/Smad pathway is one of the most extensively characterized profibrotic signaling axes in cardiac fibrosis. Under pathological conditions such as MI, pressure overload, and metabolic cardiomyopathy, TGF-β signaling is activated within injured and remodeling myocardium and contributes to tissue repair, ECM remodeling, and progressive fibrosis [39].

Canonical TGF-β signaling begins when TGF-β binds to its transmembrane receptor complex. The type II receptor subsequently activates the type I receptor, leading to phosphorylation of Smad2 and Smad3. Phosphorylated Smad2/3 associates with Smad4 and translocates to the nucleus, where the resulting complex regulates transcription of profibrotic target genes [40]. In the fibrotic heart, this transcriptional program promotes fibroblast activation, myofibroblast differentiation, and excessive interstitial deposition of type I and type III collagen. These changes increase myocardial stiffness and contribute to adverse structural remodeling [41]. Although TGF-β also activates several noncanonical pathways, current evidence identifies Smad3 as a major downstream mediator of its profibrotic actions in the heart [42].

Further studies indicate that cardiac fibroblasts are major cellular effectors through which TGF-β signaling promotes fibrotic remodeling [43]. In obese diabetic mice, fibroblast-specific activation of TGF-β/Smad3 signaling contributes directly to cardiac dysfunction, fibrosis, and myocardial hypertrophy. TGF-β signaling, however, is not uniformly pathogenic. Basal pathway activity may help maintain normal diastolic function, whereas excessive activation in obesity and diabetes can promote adverse remodeling. Its biological effects therefore depend on disease context, the magnitude of pathway activation, and fibroblast phenotype. Endogenous regulatory mechanisms can also restrain this response. For example, induction of Smad7 in fibroblasts prevents the development of cardiac remodeling under pressure-overload conditions [44]. The consequences of TGF-β signaling thus depend not only on pathway activation but also on the timing and effectiveness of mechanisms that limit its activity.

The profibrotic effects of TGF-β signaling extend beyond canonical transcriptional regulation. Chromatin-level analyses have shown that TGF-β stimulation promotes nuclear localization of ATP citrate lyase (ACLY), enhances its interaction with SMAD2/3, and increases H3K27ac enrichment at fibrosis-associated gene loci. These epigenetic alterations may contribute to the establishment and persistence of the myofibroblast phenotype [45]. TGF-β/Smad signaling should therefore be viewed not only as an upstream initiator of fibrotic responses but also as a mechanism that reinforces and stabilizes the fibrotic cellular state.

### RAAS and neurohumoral signaling

Among the neurohumoral pathways implicated in cardiac fibrosis, the renin–angiotensin–aldosterone system (RAAS) is one of the most extensively studied profibrotic axes and has particularly direct clinical relevance to anti-remodeling therapy [46, 47]. Cardiac RAAS activation occurs in pathological conditions such as MI, pressure overload, and chronic heart failure [48, 49]. Within the fibrotic myocardium, angiotensin II (Ang II) serves as a major effector of RAAS, whereas aldosterone acts as an important downstream mediator that further amplifies profibrotic responses. Ang II and aldosterone exert their effects predominantly through the angiotensin II type 1 receptor (AT1R) [50] and mineralocorticoid receptor (MR) [51], in cardiac fibroblasts. Activation of these signaling pathways promotes fibroblast activation, ECM deposition, and adverse cardiac remodeling [52]. Ang II drives fibrosis through several downstream mechanisms. Through AT1R/Gαq-dependent signaling, including activation of the TGF-β1/ERK pathway, Ang II promotes fibroblast-to-myofibroblast differentiation [53, 54]. Recent evidence further indicates that the Ang II-induced fibrotic transcriptional program can be amplified by transcriptional regulatory axes such as IRX2-EGR1 [55]. The aldosterone/MR axis likewise promotes cardiac fibroblast proliferation and enhances inflammation-associated remodeling [56]. Importantly, its profibrotic effects extend beyond fibroblasts. MR signaling contributes to cardiac fibrosis and remodeling in multiple cell types, including macrophages [57], T cells, and cardiomyocytes [58–60]. RAAS signaling is not restricted to the classical ACE/Ang II/AT1R profibrotic axis. Increasing attention has focused on counter-regulatory pathways, particularly the ACE2/Ang-(1–7)/Mas axis and signaling mediated by the angiotensin II type 2 receptor (AT2R). These pathways can partially oppose hypertrophic, inflammatory, and fibrotic responses, highlighting RAAS as a dynamically regulated network comprising both pathogenic and protective branches [61].

The profibrotic effects of RAAS are also closely interconnected with activation of the sympathetic nervous system (SNS). In heart failure, sympathetic activation stimulates renin release, whereas excessive RAAS activity can in turn sustain sympathetic excitation. This bidirectional interaction creates a self-reinforcing cycle that promotes inflammation, oxidative stress, and progressive structural remodeling [46].

### Mechanical stress and mechanotransduction

Mechanical stress is a major physical component of the cardiac fibrotic microenvironment. Its profibrotic effects depend largely on the ability of cardiac fibroblasts to sense and respond to tensile forces and changes in ECM stiffness [62, 63]. Tensile stress and matrix stiffening are detected through mechanosensitive structures, including integrin-based adhesion complexes, mechanosensitive ion channels, and the cytoskeleton. The resulting signaling responses promote fibroblast activation, myofibroblast differentiation, and ECM deposition.

Integrins are among the principal mechanosensors linking the ECM to the intracellular cytoskeleton [64]. Fibroblast-surface integrins, including β1- and αv-containing integrins, transmit extracellular mechanical forces to the actin cytoskeleton through focal adhesion proteins such as talin, vinculin, and focal adhesion kinase (FAK). This force transmission activates downstream pathways involving Src and RhoA/ROCK, thereby promoting stress-fiber formation and increasing cellular contractility [63]. Certain αv integrins also facilitate the local activation of latent TGF-β, directly coupling mechanical stimulation to canonical profibrotic signaling and amplifying myofibroblast differentiation and ECM deposition [65, 66]. Integrins therefore function not only as mechanical anchors but also as signaling platforms that integrate physical forces with growth factor-mediated responses.

Yes-associated protein (YAP) and transcriptional coactivator with PDZ-binding motif (TAZ) are major transcriptional regulators of cellular responses to mechanical stress. Increased matrix stiffness, mechanical loading, and cytoskeletal tension can alter the subcellular localization and transcriptional activity of YAP/TAZ, thereby regulating genes involved in proliferation, contractility, and matrix remodeling. In a human three-dimensional model of cardiac fibrosis, TGF-β1-induced myofibroblast activation and matrix stiffening were associated with activation of YAP/Hippo-related transcriptional signaling [67]. In a MI model, fibroblast-specific deletion of Yap/Taz reduced fibrosis and inflammatory responses and improved cardiac function [68]. Similarly, inhibition of YAP activity in fibroblasts attenuated cardiac fibrosis and dysfunction through modulation of myocardin-related transcription factor A (MRTF-A)-associated transcriptional programs [69]. The profibrotic influence of YAP/TAZ is not confined to direct regulation of fibroblast phenotype. These factors may also contribute indirectly to cardiac fibrosis by modulating cardiomyocyte adaptation to pressure overload [70, 71], epicardial inflammatory activation, and intercellular paracrine signaling [72]. Together, these findings indicate that YAP/TAZ function as integrators of mechanical and multicellular signals that contribute to the progression of mechanically driven cardiac fibrosis.

In addition to the integrin-cytoskeleton-YAP/TAZ axis, mechanosensitive ion channels, particularly members of the transient receptor potential (TRP) family, participate in cardiac fibroblast mechanotransduction. Mechanosensitive Ca^2+^ channels such as transient receptor potential vanilloid 4 (TRPV4) integrate changes in matrix stiffness with soluble profibrotic cues, including TGF-β1, thereby promoting fibroblast-to-myofibroblast differentiation [73]. Non-TRP mechanosensitive channels, including Piezo1, have also emerged as important regulators of fibroblast responses to mechanical stress [74]. In cardiac fibroblasts, Piezo1 activation induces Ca^2+^ influx, activates p38 MAPK signaling, and promotes IL-6 secretion [75]. These observations suggest that mechanical stimulation regulates not only fibroblast contraction and differentiation but also the amplification of fibrotic responses through inflammatory paracrine signaling [76].

### Inflammatory and immune pathways

Following myocardial injury, inflammatory and immune responses actively shape the subsequent fibrotic response. Cytokines, chemokines, and damage-associated mediators released during tissue injury can act directly on cardiac fibroblasts and promote fibrosis by regulating fibroblast activation, immune-cell recruitment, and ECM deposition [77, 78].

Nuclear factor-κB (NF-κB) is a key transcriptional regulator of inflammatory responses. It controls the expression of numerous cytokines and chemokines, facilitates recruitment of inflammatory cells such as monocytes and macrophages, and amplifies local inflammatory signaling [79]. Fibroblast-specific deletion of IκB kinase β reduces Ang II-induced proinflammatory and profibrotic responses in cardiac fibroblasts [80]. In pressure-overload models, NF-κB activation in cardiac fibroblasts also promotes C–C motif chemokine ligand 2 (CCL2) expression and recruitment of Ly6C^hi^ monocytes [81]. These findings identify fibroblast-intrinsic NF-κB signaling as an important link between inflammation and fibrosis. The NLR family pyrin domain containing 3 (NLRP3) inflammasome functions as an inflammatory amplifier and effector complex and is closely associated with mitochondrial dysfunction and ROS accumulation [82, 83]. Once activated, NLRP3 promotes caspase-1-dependent maturation and release of interleukin-1β (IL-1β) and IL-18, reinforcing local inflammatory responses and creating conditions that favor fibrotic remodeling. In experimental models of MI, pharmacological inhibition of NLRP3 with MCC950 [84] or oridonin [85] attenuated cardiac fibrosis and adverse remodeling.

Among proinflammatory cytokines, tumor necrosis factor-α (TNF-α) and IL-6 are representative mediators with well-established roles in myocardial inflammation and fibrotic remodeling. Elevated TNF-α levels can amplify inflammatory responses and contribute to ventricular remodeling and cardiac dysfunction [86–88]. IL-6 promotes cardiac fibroblast activation and myofibroblast differentiation [89] and is also associated with increased collagen deposition, greater ventricular stiffness, and diastolic dysfunction [90]. Studies in patients with dilated cardiomyopathy have further shown that circulating TNF-α and IL-6 levels correlate with markers of collagen metabolism and left atrial enlargement. These findings suggest that proinflammatory cytokines contribute to dysregulated collagen turnover and structural remodeling of the myocardial interstitium [91].

Chemokines facilitate immune-cell recruitment and amplify local inflammation during the transition from inflammatory injury to fibrosis. Among these pathways, the CCL2/MCP-1-CCR2 axis is particularly well characterized [92]. Consistent with this, human myocardial studies indicate that CCR2 + cardiac macrophages are maintained in part through monocyte recruitment and exhibit inflammatory properties [93]. Bidirectional crosstalk between macrophages and fibroblasts is central to this process [32]. Activated macrophages release mediators such as TGF-β, platelet-derived growth factor (PDGF), IL-1β, CCL2, osteopontin, and galectin-3, which regulate fibroblast proliferation, migration, and acquisition of profibrotic phenotypes [94]. Conversely, activated fibroblasts secrete chemokines and inflammatory mediators that sustain immune-cell recruitment and shape immune-cell phenotypes. This reciprocal interaction establishes a self-reinforcing inflammation–fibrosis feedback loop. Macrophages of different developmental origins and functional states make distinct contributions to this response. Recruited CCR2^+^ macrophages generally exhibit proinflammatory and pro-remodeling properties, whereas subsets of resident macrophages may contribute to tissue homeostasis and coordinated repair [95].

TGF-β/Smad signaling, RAAS activation, mechanical stress, and inflammatory mediators can collectively amplify cellular oxidative stress, disturb calcium and redox homeostasis, and increase metabolic demand. Persistent activation of these pathways can impair mitochondrial respiratory-chain function and quality control, thereby promoting mitochondrial reactive oxygen species (ROS) accumulation and altered substrate utilization. Mitochondrial dysfunction can, in turn, reinforce inflammatory and profibrotic signaling, forming a bidirectional feedback loop between classical profibrotic pathways and intracellular redox and metabolic remodeling. Mitochondria therefore represent an important mechanistic interface through which extracellular profibrotic stimuli are integrated into sustained cellular responses.

### Mitochondrial dysfunction, metabolic remodeling, and SIRT3-mediated regulation

Mitochondrial dysfunction during cardiac remodeling is primarily characterized by impaired energy production and excessive ROS generation and is frequently accompanied by metabolic reprogramming [96–98]. Changes in mitochondrial metabolism can alter NAD⁺ availability and the NAD⁺/NADH balance and are associated with changes in mitochondrial protein acetylation. Because protein acetylation is reversible, the balance between acetylation and deacetylation is important for regulating mitochondrial protein function [2, 4]. SIRT3 is a major mitochondrial NAD⁺-dependent deacetylase that removes acetyl groups from multiple mitochondrial proteins [4]. In experimental cardiac models, this activity may help preserve mitochondrial function. However, SIRT3 expression, activity, and downstream effects may vary according to disease context and stage.

#### Oxidative stress and mitochondrial dysfunction

Following myocardial injury, mitochondria can become a major source of excessive ROS. Pathological stimuli such as MI, pressure overload, diabetes, and aging can impair mitochondrial respiratory-chain function, causing ROS production to exceed the capacity of endogenous antioxidant systems [96–98].

Excessive ROS not only reflect cellular oxidative injury but also function as signaling mediators that promote fibrotic responses. In cardiac fibroblasts, elevated ROS levels enhance fibroblast activation and regulate collagen synthesis and matrix remodeling [99]. NOX4-derived ROS, for example, contribute to TGF-β1-induced myofibroblast differentiation [100]. Mitochondrial dysfunction can further sustain profibrotic signaling by promoting ROS accumulation, impairing oxidative phosphorylation, and disrupting mitochondrial quality-control mechanisms [101]. These abnormalities also disturb cellular energy balance and redox homeostasis, thereby altering substrate utilization and metabolic signaling during fibrotic remodeling.

#### Metabolic reprogramming

The adult heart derives most of its energy from fatty acid oxidation (FAO), which accounts for approximately 60–90% of adenosine triphosphate (ATP) production, whereas glucose oxidation and lactate utilization together contribute approximately 20–30% of cardiac energy supply. Under pathological conditions, however, patterns of substrate utilization and energy metabolism undergo substantial remodeling [102–104].

Cardiac fibroblasts have relatively low metabolic demands under resting conditions. Following injury, however, transition to the myofibroblast state requires increased energy production and biosynthetic substrate availability to support proliferation, migration, cytoskeletal remodeling, α-smooth muscle actin (α-SMA) expression, and collagen synthesis [105, 106]. Accordingly, cardiac fibroblast activation is frequently accompanied by increased glycolytic activity. Following MI, inhibition of glycolysis attenuates cardiac fibroblast activation and fibrosis. Similarly, TGF-β1 enhances glycolysis in cardiac fibroblasts and promotes α-SMA and collagen expression through key glycolytic regulators such as PFKFB3 [107, 108].

Fibrosis-associated metabolic remodeling extends beyond fibroblasts and involves the broader cardiac metabolic network. Impaired FAO can reduce ATP production while promoting the accumulation of lipid intermediates, thereby increasing metabolic stress. Defective branched-chain amino acid (BCAA) catabolism is another characteristic metabolic alteration in the failing heart, whereas restoration of BCAA catabolism or enhancement of BCAA oxidation can improve cardiac function [109, 110]. The failing heart can increase ketone-body utilization as an alternative energy source [111]. In pressure-overloaded failing hearts, increasing β-hydroxybutyrate availability enhances ketone oxidation and overall energy production, although cardiac efficiency is not improved [112]. More broadly, metabolic reprogramming affects not only substrate selection and ATP production but also intracellular energy sensing and mitochondrial quality control. Changes in the AMP/ATP ratio can activate AMPK and subsequently modulate mTOR–ULK1-dependent autophagy [113]. Likewise, alterations in the NAD^+^/NADH ratio can influence NAD^+^-dependent deacetylation and thereby modify the reversible acetylation of mitochondrial proteins [114]. Changes in NAD^+^ availability may therefore provide an important mechanistic link between metabolic remodeling and SIRT3-dependent mitochondrial deacetylation during cardiac fibrosis.

#### SIRT3-mediated regulation of mitochondrial deacetylation

**Fig. 2:**
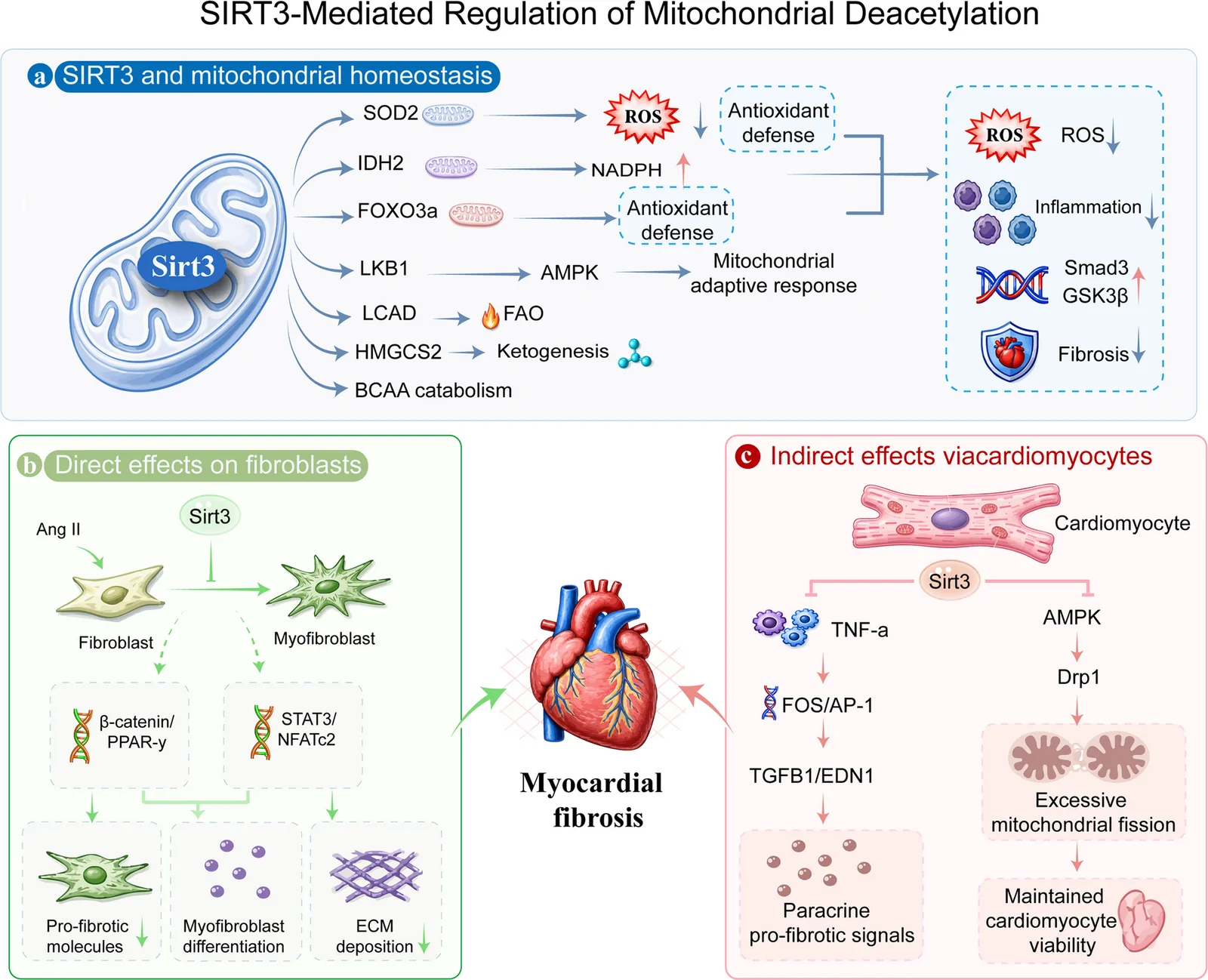
SIRT3-associated mechanisms in myocardial fibrosis. Schematic overview of SIRT3-associated mechanisms implicated in myocardial fibrotic remodeling. Panel **a** summarizes SIRT3-related regulation of mitochondrial homeostasis, redox balance, metabolic adaptation, and stress responses. Panel **b** illustrates SIRT3-associated regulation of fibroblast activation and myofibroblast differentiation reported in experimental models. Panel **c** summarizes cardiomyocyte-mediated mechanisms involving profibrotic paracrine signaling, mitochondrial dynamics, and cardiomyocyte stress responses. These effects are context-dependent and may vary according to disease model, cell type, and stage of remodeling. Abbreviations: AMPK, AMP-activated protein kinase; Ang II, angiotensin II; AP-1, activator protein-1; BCAA, branched-chain amino acid; Drp1, dynamin-related protein 1; ECM, extracellular matrix; EDN1, endothelin 1; FAO, fatty acid oxidation; FOXO3a, forkhead box O3a; GSK3β, glycogen synthase kinase 3β; HMGCS2, mitochondrial 3-hydroxy-3-methylglutaryl-CoA synthase 2; IDH2, isocitrate dehydrogenase 2; LCAD, long-chain acyl-CoA dehydrogenase; LKB1, liver kinase B1; NADPH, reduced nicotinamide adenine dinucleotide phosphate; NFATc2, nuclear factor of activated T cells 2; PPAR-γ, peroxisome proliferator-activated receptor-γ; ROS, reactive oxygen species; SIRT3, sirtuin 3; SOD2, superoxide dismutase 2; STAT3, signal transducer and activator of transcription 3; TGFB1, transforming growth factor beta 1; TNF-α, tumor necrosis factor-α

The role of SIRT3 in cardiac fibrosis appears to vary across pathological contexts rather than being uniform across disease states. Available evidence indicates that SIRT3 expression and activity may depend on the initiating injury, underlying metabolic milieu, and stage of disease. During post-infarction remodeling, SIRT3 expression has been reported to decline with disease progression, whereas restoration of SIRT3 activity is associated with reduced mitochondrial fission and attenuated fibrosis. Under pressure overload, SIRT3 deficiency is associated with greater cardiac hypertrophy and fibrosis, impaired mitochondrial energetics, and more pronounced contractile dysfunction [115]. In diabetic cardiomyopathy, reduced myocardial SIRT3 expression has been associated with mitochondrial injury, oxidative stress, necroptosis, and NLRP3 inflammasome activation [116, 117]. In aging models, SIRT3 deficiency predisposes the heart to hypertrophy and fibrosis and increases its vulnerability to pressure overload [118]. Direct evidence regarding the role of SIRT3 in HFpEF remains limited. Collectively, these observations support a context-dependent role for SIRT3 in cardiac remodeling (Fig. 2).

At the mechanistic level, SIRT3-dependent deacetylation acts on multiple mitochondrial substrates involved in oxidative stress, energy metabolism, and mitochondrial homeostasis (Table 1).

**Table 1:** SIRT3-associated molecular pathways and effector mechanisms in cardiac fibrosis

Functional level	Core molecular pathway	Mechanism of action	Main cellular/experimental evidence	References
Redox regulation	SIRT3-SOD2/IDH2	Deacetylation-mediated activation of antioxidant enzymes	Cardiomyocytes	[119, 120]
Antioxidant and stress-response regulation	SIRT3–FOXO3a/LKB1–AMPK	Regulation of antioxidant defense and adaptive mitochondrial responses	Cardiomyocytes	[121]
Fibroblast profibrotic signaling	SIRT3–β-catenin/PPAR-γ/STAT3–NFATc2	Regulation of fibroblast-to-myofibroblast transdifferentiation	Cardiac fibroblasts	[122, 123]
Metabolic adaptation	SIRT3–LCAD/HMGCS2	Regulation of fatty acid oxidation and ketone body metabolism through deacetylation	Metabolic enzyme studies	[124–126]
Immune microenvironment	SIRT3-ROS-NLRP3 NF-κB-MCP-1	Regulation of inflammasome activation and inflammatory signaling	Cardiac models	[116, 127]
Mitochondrial dynamics	SIRT3-AMPK-Drp1	Inhibition of excessive mitochondrial fission after myocardial infarction	Cardiomyocytes	[128]

*Abbreviations:* *AMPK* AMP-activated protein kinase, *Drp1* dynamin-related protein 1, *FOXO3a* forkhead box O3a, *HMGCS2* mitochondrial 3-hydroxy-3-methylglutaryl-CoA synthase 2, *IDH2* isocitrate dehydrogenase 2, *LCAD* long-chain acyl-CoA dehydrogenase, *LKB1* liver kinase B1, *MCP-1* monocyte chemoattractant protein-1, *NF-κB* nuclear factor-κB, *NFATc2* nuclear factor of activated T cells 2, *NLRP3* NLR family pyrin domain containing 3, *PPAR-γ* peroxisome proliferator-activated receptor-γ, *ROS* reactive oxygen species, *SIRT3* sirtuin 3, *SOD2* superoxide dismutase 2, *STAT3* signal transducer and activator of transcription 3

SIRT3 deacetylates and activates SOD2 [119] and IDH2 [120], thereby enhancing ROS scavenging and NADPH production and limiting persistent oxidative stress. SIRT3 also regulates FOXO3a [129] and LKB1-AMPK signaling [121, 130], contributing to antioxidant defense and adaptive mitochondrial responses. In addition, deacetylation of metabolic enzymes such as LCAD [124] and HMGCS2 [125] links SIRT3 activity to fatty acid oxidation, ketone-body metabolism, and metabolic adaptation under pathological stress. SIRT3 signaling may also modulate inflammatory responses through ROS–NF-κB–MCP-1 and FOS/AP-1-related pathways [127, 131]. Through pathways including GSK3β-Smad3 signaling, SIRT3 has further been implicated in the regulation of profibrotic transcriptional programs and fibroblast activation [132].

Current evidence suggests that SIRT3 modulates cardiac fibrosis through both fibroblast-intrinsic and cardiomyocyte-mediated mechanisms. In cardiac fibroblasts, SIRT3 regulates fibroblast-to-myofibroblast transdifferentiation in response to profibrotic stimuli. Under Ang II stimulation, SIRT3 deficiency enhances fibroblast-to-myofibroblast transition and increases the expression of fibrotic mediators. These effects have been linked to β-catenin/PPAR-γ [122] and STAT3-NFATc2 signaling [123].

Early studies identified SIRT3 as a stress-responsive deacetylase in cardiomyocytes and demonstrated that increased SIRT3 expression protects these cells from genotoxic- and oxidative stress-induced cell death [133]. Cardiomyocyte-specific deletion of SIRT3 leads to age-dependent cardiac fibrosis [126]. In cardiomyocyte models, SIRT3 overexpression attenuates TNF-α-induced FOS expression and activator protein-1 (AP-1) activity while reducing downstream profibrotic mediators, including TGFB1 and EDN1 [131]. Following MI, SIRT3 overexpression suppresses excessive mitochondrial fission and preserves cardiomyocyte viability through the AMPK-Drp1 pathway, accompanied by reduced cardiac fibrosis [128].

## ECM synthesis, degradation, and cross-linking

Profibrotic signaling disrupts ECM homeostasis. Cardiac fibrosis involves a coordinated sequence of matrix alterations, including increased ECM synthesis, impaired degradation, enhanced collagen cross-linking, and progressive matrix stiffening [134]. These changes not only determine scar formation and tissue stability but also influence myocardial compliance, electrical conduction, and ventricular remodeling.

### ECM synthesis and accumulation

In the healthy heart, the ECM provides an essential structural and biochemical microenvironment that supports mechanical stability, cell adhesion, signal transduction, and cardiomyocyte alignment [135–137]. Under homeostatic conditions, cardiac fibroblasts maintain continuous but relatively low-level ECM turnover. Pathological stress, however, can drive fibroblasts toward a matrix-producing phenotype.

Following acute myocardial injury, particularly MI, increased ECM synthesis initially serves an essential reparative function [138]. Lineage-tracing studies have demonstrated that periostin-positive myofibroblasts in the injured heart arise predominantly from tissue-resident Tcf21-lineage fibroblasts [6, 26]. Depletion of these cells reduces collagen production and scar formation within the infarcted region but increases the risk of ventricular wall rupture. These findings demonstrate that fibroblast-derived ECM production is required for effective myocardial repair and maintenance of structural integrity after injury.

When matrix synthesis fails to resolve appropriately after injury, or is repeatedly stimulated by conditions such as pressure overload, hypertensive heart failure, and chronic ischemic cardiomyopathy, ECM turnover can shift from dynamic equilibrium toward persistent net accumulation. An endomyocardial biopsy study in patients with hypertensive heart failure demonstrated increased myocardial collagen content, enhanced type I collagen deposition, and elevated markers of type I collagen synthesis compared with controls [139]. These indices were also higher than those observed in hypertensive patients without heart failure. Similarly, quantitative proteomic analysis of myocardial tissue from patients with chronic ischemic cardiomyopathy revealed increased abundance of type I and type III collagens, suggesting that fibrillar collagen accumulation may further reinforce pathological ECM deposition [140].

### Imbalance in ECM degradation

Pathological ECM accumulation results not only from increased matrix synthesis but also from impaired matrix degradation and turnover. The matrix metalloproteinase/tissue inhibitor of metalloproteinase (MMP/TIMP) system is a major regulatory axis controlling cardiac ECM degradation and remodeling [141, 142]. Following myocardial injury, this system undergoes rapid reorganization, shifting matrix turnover away from physiological renewal toward either excessive degradation or pathological stabilization.

During acute MI, excessive ECM degradation is an important early component of ventricular remodeling. Early studies showed that inhibition of plasminogen activators or MMPs can reduce the incidence of post-infarction cardiac rupture, although such interventions may also impair angiogenesis and compromise cardiac recovery [143]. Individual MMPs exert distinct effects during this process. Deletion of MMP-9 reduces left ventricular dilation and abnormal collagen accumulation after experimental MI [144]. More recent preclinical work showed that sustained local delivery of the MMP-9 inhibitory protein C9 improved cardiac function and ventricular geometry after MI [145]. Targeted deletion or pharmacological inhibition of MMP-2 also reduces cardiac rupture following acute MI [146] and delays subsequent remodeling by preserving ECM components such as laminin and fibronectin while limiting macrophage infiltration. In human myocardial tissue obtained from patients with infarct rupture, both the abundance and activity of MMP-8 and MMP-9 were substantially higher than in non-ruptured infarct tissue and were accompanied by more pronounced inflammatory-cell infiltration. Excessive local MMP activation after acute MI may therefore accelerate ECM degradation within the infarcted region, destabilize the matrix scaffold, and increase mechanical vulnerability of the ventricular wall [147].

By contrast, chronic cardiac fibrosis more commonly reflects a combination of enhanced ECM synthesis and inadequate degradation. Studies of human hearts exposed to chronic pressure overload have shown that the MMP/TIMP balance shifts toward TIMP predominance, a pattern associated with interstitial fibrosis and diastolic dysfunction [148]. The functional effects of individual TIMP family members nevertheless vary according to pathological context and disease stage. TIMP-1 deficiency exacerbates left ventricular remodeling after MI in mice [149]. In Ang II-induced or pressure-overload models, however, TIMP-1 has also been shown to promote collagen synthesis and deposition through mechanisms involving CD63-integrin β1 signaling [150].

### ECM cross-linking and matrix stiffening

ECM accumulation and impaired degradation alone do not fully account for the functional consequences of cardiac fibrosis. Alterations in matrix architecture and mechanical properties are equally important. Following synthesis and secretion, collagen molecules undergo fibril assembly, maturation, and cross-linking to form a stable three-dimensional matrix network. Excessive cross-linking converts relatively soluble and degradable collagen into insoluble fibers that are more resistant to proteolytic degradation. This process increases matrix rigidity, reduces tissue elasticity, and elevates passive myocardial stiffness [151–153].

The lysyl oxidase family (LOX/LOXLs) constitutes a major enzymatic system responsible for covalent cross-linking of collagen and elastin [154]. Following MI, several LOX family members, including LOX and LOXL1-4, are markedly upregulated in association with accumulation of mature collagen fibers within the infarcted region [155]. Non-enzymatic cross-linking mediated by advanced glycation end products (AGEs) may also contribute substantially to myocardial stiffening in diabetes, aging, and metabolic disease. In an aged diabetic dog model, the AGE cross-link breaker ALT-711 reduced aortic stiffness, reversed increases in myocardial collagen, and improved left ventricular function [156]. Beyond AGE-mediated cross-linking, transglutaminases may also contribute to collagen stabilization. In vitro studies using neonatal rat ventricular fibroblasts showed that knockdown of TG1 or TG2 reduced insoluble collagen accumulation and decreased the collagen cross-linking ratio [157].

Matrix stiffening is not merely a consequence of fibrotic remodeling but also acts as a mechanical stimulus that further reinforces fibroblast activation [158]. A stiffened ECM increases intracellular tension through mechanotransduction pathways, thereby promoting α-SMA expression, stress-fiber formation, and additional matrix synthesis. In vitro models with controlled substrate stiffness have shown that rigid matrices activate YAP signaling in cardiac fibroblasts and promote their transition toward an activated phenotype [159]. Three-dimensional hydrogel systems similarly demonstrate that matrix stiffening promotes fibroblast-to-myofibroblast differentiation, whereas matrix softening can partially reverse this phenotypic transition. Integrin β1 signaling and focal adhesion formation contribute to these stiffness-dependent responses [160].

## Translational advances and therapeutic strategies

Research on cardiac fibrosis has progressively expanded from mechanistic investigation toward clinical assessment and therapeutic intervention. Molecular and cellular findings are increasingly being translated into approaches for disease detection, risk stratification, and treatment development. Circulating biomarkers, advanced imaging techniques, and integrated stratification tools have improved the clinical assessment of fibrotic remodeling, while therapeutic strategies targeting ECM turnover, metabolic dysfunction, and inflammatory signaling are being explored as potential antifibrotic interventions.

### Diagnosis and prognosis assessment

Because cardiac fibrosis is biologically heterogeneous and varies over time and across myocardial regions, no single diagnostic modality can fully characterize its onset, distribution, and progression. Clinical assessment therefore increasingly relies on the integration of circulating biomarkers, imaging features, and multidimensional risk-stratification approaches. Such multimodal assessment may improve the detection of cardiac fibrosis while providing a more clinically interpretable estimate of fibrotic burden and disease activity [161, 162] (Table 2).

**Table 2:** Diagnostic and prognostic tools for cardiac fibrosis

Tool/indicator	Main feature assessed	Strengths	Limitations	References
PICP/PIIINP/CITP/MMP-1	Collagen synthesis/degradation/crosslinking	Repeatable; dynamic monitoring	Low cardiac specificity; weak correlation with true fibrosis burden	[139, 152, 163, 164]
Galectin-3/sST2	Matrix remodeling; inflammation; myocardial stress	Easily accessible	Affected by systemic inflammation and renal function	[164–167]
LGE-CMR	Focal replacement fibrosis; infarct scar	Accurate focal detection	Limited for diffuse interstitial fibrosis; insensitive to early lesions	[168]
T1/ECV/synthetic ECV	Diffuse interstitial fibrosis; extracellular matrix expansion	Useful for early and diffuse changes	Affected by edema, inflammation, fat/iron deposition, and platform differences	[169]
Myocardial strain imaging	Subclinical mechanical dysfunction	Detects dysfunction before structural change	Load- and image quality-dependent	[170]
FAPI-PET	Activated fibroblast activity	Detects active fibrotic lesions	Limited standardization; costly; low availability	[171–175]
Radiomics/AI-assisted imaging analysis	Hidden imaging patterns	Improves diagnosis and risk stratification	Limited validation; poor standardization	[176, 177]
Endomyocardial biopsy	Direct histologic evidence; fibrosis type/severity	Direct histological gold standard	Invasive; sampling error; unsuitable for routine follow-up	[178]

*Abbreviations:* *AI* artificial intelligence, *CITP* carboxy-terminal telopeptide of type I collagen, *CMR* cardiovascular magnetic resonance, *ECV* extracellular volume fraction, *FAPI* fibroblast activation protein inhibitor, *LGE* late gadolinium enhancement, *MMP-1* matrix metalloproteinase-1, *PET* positron emission tomography, *PICP* carboxy-terminal propeptide of type I procollagen, *PIIINP* amino-terminal propeptide of type III procollagen, *sST2*, soluble suppression of tumorigenicity 2

#### Circulating biomarkers and hematological parameters

Circulating biomarkers are clinically attractive because they are readily accessible, can be measured serially, and may reflect active fibrotic remodeling before overt structural abnormalities become apparent. The most extensively studied candidates are markers of collagen metabolism, including the carboxy-terminal propeptide of type I procollagen (PICP), the amino-terminal propeptide of type III procollagen (PIIINP), and the ratio of the carboxy-terminal telopeptide of type I collagen to matrix metalloproteinase-1 (CITP/MMP-1) [139, 152, 163]. These biomarkers provide indirect information regarding collagen synthesis, degradation, and cross-linking. However, their correlations with the actual extent of myocardial fibrosis remain variable across studies [164]. Circulating markers such as galectin-3 [165] and soluble ST2 (sST2) [166] have also been investigated in the context of cardiac fibrosis. In addition to their associations with matrix remodeling, these biomarkers reflect inflammatory signaling and myocardial stress. They may therefore help identify patients with active fibrotic remodeling and an unfavorable prognosis, particularly among those with heart failure or cardiomyopathy.

A major limitation of most circulating biomarkers is their lack of cardiac specificity. Their concentrations can be influenced by fibrosis in other organs, systemic inflammation, metabolic disease, renal dysfunction, and other comorbid conditions [161, 162, 167].

#### Imaging assessment and emerging stratification tools

Imaging plays a central role in the noninvasive evaluation of cardiac fibrosis. Cardiovascular magnetic resonance (CMR) is currently the principal imaging modality for myocardial tissue characterization [168]. Two approaches are particularly important. Late gadolinium enhancement (LGE) is well suited to the detection of focal replacement fibrosis and established scar tissue, whereas extracellular volume fraction (ECV) provides a more quantitative assessment of diffuse interstitial expansion and may detect abnormalities before distinct LGE patterns become apparent. ECV correlates closely with histological measures of ECM expansion and is generally less dependent on acquisition conditions than native T1 values alone. Conventional ECV calculation, however, requires contemporaneous measurement of hematocrit. Simplified approaches such as synthetic ECV have therefore been developed to improve clinical feasibility [179].

Beyond CMR, strain-based imaging provides complementary functional information. Myocardial strain does not directly quantify fibrosis [170], but it can detect subtle mechanical abnormalities associated with fibrotic remodeling and may therefore facilitate early phenotypic identification and prognostic stratification. Fibroblast activation protein (FAP)-targeted positron emission tomography using fibroblast activation protein inhibitor (FAPI) tracers offers a different approach by enabling visualization of activated fibroblasts in vivo [171–174]. High-dimensional feature-extraction strategies based on radiomics and artificial intelligence may further improve risk stratification by integrating complex imaging phenotypes [176, 177]. A combined PET/CMR study using gallium-68-labeled fibroblast activation protein inhibitor ([^68^ Ga]FAPI) demonstrated that myocardial FAPI uptake after acute MI peaked approximately 1–2 weeks after symptom onset and extended beyond the scar region defined by LGE. Baseline FAPI uptake was also associated with subsequent left ventricular remodeling and scar burden at 1 year [175].

Histological assessment remains the reference standard for quantifying cardiac fibrosis. However, endomyocardial biopsy is invasive, susceptible to sampling error, and unsuitable for routine longitudinal monitoring [178]. Circulating biomarkers are more readily obtained but lack cardiac specificity, while imaging modalities also have important limitations. LGE has relatively low sensitivity for diffuse interstitial fibrosis and requires administration of gadolinium-based contrast agents. Native T1 mapping and ECV expand the quantitative assessment of diffuse myocardial abnormalities, but their values can be influenced by edema, infiltrative disease, fat or iron deposition, scanner platform, acquisition sequence, and institution-specific reference ranges [169].

#### Clinical value and limitations of SIRT3 as a potential biomarker

Patients with cardiovascular diseases, particularly hypertension and dilated cardiomyopathy, have been reported to exhibit lower SIRT3 levels than healthy controls, supporting its potential as a candidate cardiovascular biomarker [180]. In experimental models of pressure overload-induced cardiac remodeling, SIRT3 expression also changes dynamically during disease progression [181]. Together, these findings suggest that reduced SIRT3 expression may be associated with greater fibrotic burden and worsening cardiac function, raising the possibility that SIRT3 could serve as an adjunctive marker for disease stratification and clinical assessment.

However, several important limitations currently preclude the use of SIRT3 as a routine clinical biomarker. First, SIRT3 is predominantly localized to mitochondria, and clinical studies evaluating its concentration in the peripheral circulation remain scarce. Standardized analytical methods and validated cutoff values have not yet been established. Second, SIRT3 expression measured in peripheral blood mononuclear cells can be influenced by dietary interventions [182] and acute inflammatory stimulation [183], complicating extrapolation from peripheral measurements to myocardial SIRT3 status. More broadly, the evidence supporting circulating SIRT3 as a biomarker remains limited and fragmented, and large prospective studies in patients with cardiac fibrosis or heart failure are lacking.

SIRT3 should therefore currently be regarded as a mechanistically informative candidate marker rather than a clinically validated biomarker. Its future clinical utility may depend on whether SIRT3-related measurements can be integrated with imaging evidence of fibrotic activity, biomarkers of collagen turnover, and metabolic phenotyping to improve risk stratification and guide therapeutic selection.

### Therapeutic strategies and translational advances

**Fig. 3:**
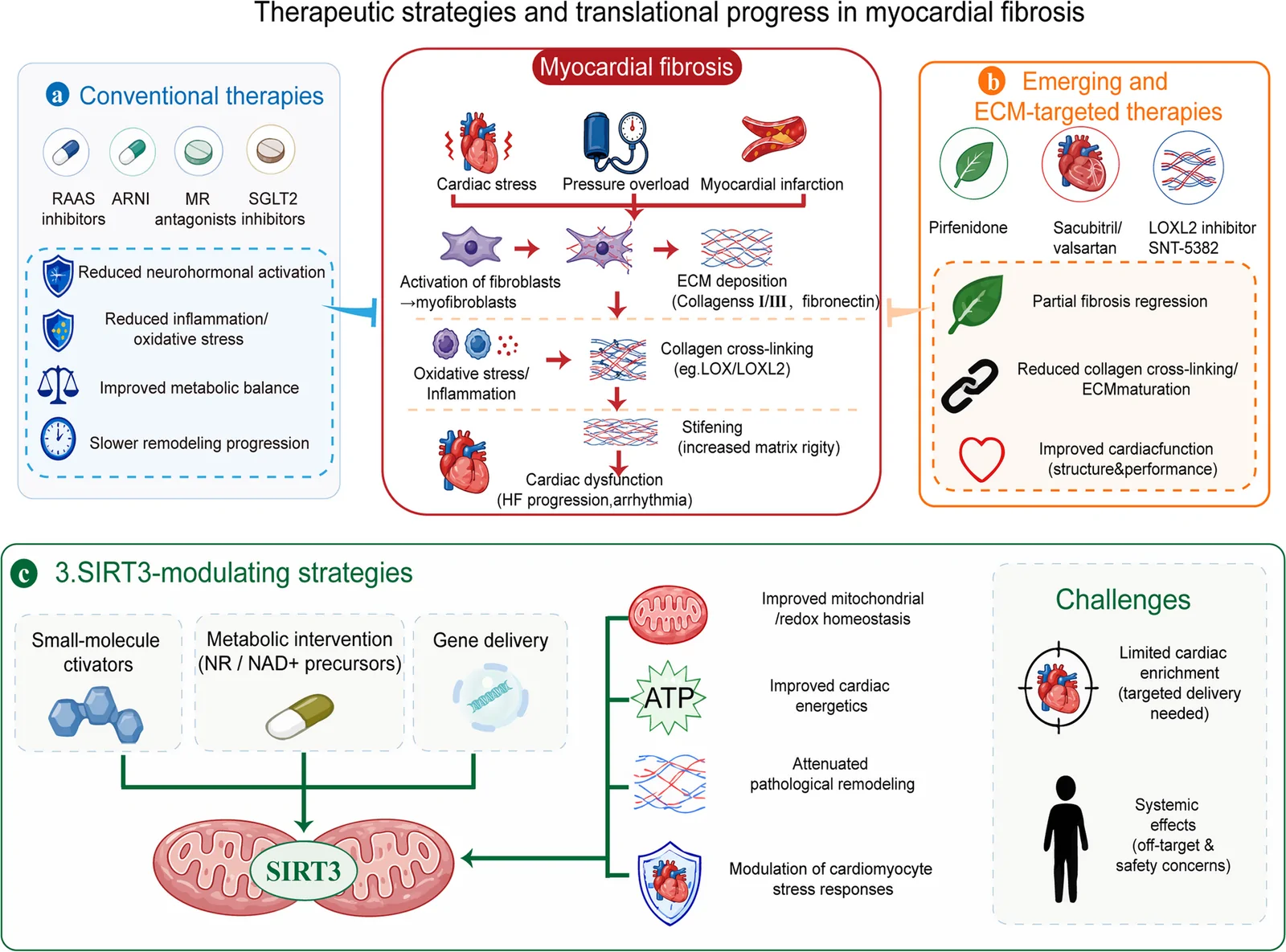
Therapeutic strategies and translational progress in myocardial fibrosis. Schematic overview of representative therapeutic approaches relevant to myocardial fibrotic remodeling. Panel **a** summarizes established cardiovascular therapies, including RAAS inhibitors, angiotensin receptor–neprilysin inhibitors (ARNIs), mineralocorticoid receptor (MR) antagonists, and sodium–glucose cotransporter 2 (SGLT2) inhibitors, which may influence fibrotic remodeling primarily through upstream neurohumoral, inflammatory, oxidative, and metabolic pathways. Panel **b** illustrates emerging and extracellular matrix (ECM)-targeted strategies, including pirfenidone, the lysyl oxidase-like 2 (LOXL2) inhibitor SNT-5382, and other approaches targeting matrix cross-linking, with fibrosis-related effects and translational evidence varying among individual strategies. Panel **c** summarizes experimental SIRT3-modulating approaches, including pharmacological modulation, NAD⁺-related metabolic interventions, and targeted delivery, together with representative effects on mitochondrial and metabolic homeostasis and major translational challenges. The type and strength of fibrosis-specific evidence differ substantially across these therapeutic strategies. Abbreviations: ARNI, angiotensin receptor–neprilysin inhibitor; ATP, adenosine triphosphate; ECM, extracellular matrix; HF, heart failure; LOXL2, lysyl oxidase-like 2; MR, mineralocorticoid receptor; NAD⁺, nicotinamide adenine dinucleotide; NR, nicotinamide riboside; RAAS, renin–angiotensin–aldosterone system; SGLT2, sodium–glucose cotransporter 2; SIRT3, sirtuin 3

Clinical management of cardiac fibrosis continues to rely largely on established therapies for heart failure and adverse cardiac remodeling [184] (Fig. 3). Among these treatments, inhibition of the RAAS has the most direct mechanistic connection to neurohumoral profibrotic signaling, although fibrosis-specific clinical evidence remains less extensive than evidence supporting its broader cardiovascular benefits. In chronic heart failure, RAAS activation increases Ang II and aldosterone signaling. Ang II exerts potent profibrotic effects predominantly through AT1R-dependent pathways [53, 185]. In cardiac fibroblasts, AT1R activation stimulates fibroblast growth, collagen synthesis, and myofibroblast differentiation through downstream pathways involving TGF-β1, Smad signaling, and ERK [53, 185]. Accordingly, angiotensin-converting enzyme inhibitors (ACEi) and angiotensin receptor blockers (ARBs) may influence fibrotic remodeling in addition to their established hemodynamic and neurohumoral effects. Direct fibrosis-specific evidence has been obtained from small human endomyocardial biopsy studies. Lisinopril treatment was associated with reductions in myocardial collagen volume fraction and hydroxyproline content in patients with hypertensive heart disease, whereas losartan was associated with regression of severe myocardial fibrosis and reduced left ventricular chamber stiffness in patients with a high baseline collagen burden [186, 187].

Aldosterone is another major downstream mediator of RAAS-dependent fibrotic remodeling. In addition to its renal actions, aldosterone acts through MRs expressed in cardiac fibroblasts, macrophages, cardiomyocytes, and vascular cells [51, 58, 59, 188]. MR activation promotes inflammatory and oxidative stress responses while enhancing fibroblast proliferation, collagen synthesis, and ECM deposition. Mineralocorticoid receptor antagonists (MRAs), including spironolactone and eplerenone, may therefore attenuate fibrotic remodeling by suppressing aldosterone-mediated profibrotic signaling. In humans, however, fibrosis-specific evidence for MRAs is derived predominantly from circulating biomarkers. In the randomized HOMAGE trial, spironolactone reduced serum PICP and the PICP/CITP ratio, whereas PIIINP was not significantly altered. A pooled analysis of randomized spironolactone trials similarly demonstrated a reduction in circulating PICP, supporting an effect on collagen synthesis and remodeling rather than providing direct evidence of histological fibrosis regression [189, 190].

Angiotensin receptor-neprilysin inhibitors (ARNIs) represent another established anti-remodeling strategy with potential effects on myocardial fibrosis. Sacubitril/valsartan combines AT1R blockade with neprilysin inhibition, thereby suppressing Ang II-AT1R signaling while augmenting natriuretic peptide activity. More direct fibrosis-related evidence has emerged from the randomized phase 2 REVERSE-LVH trial. In patients with hypertension and left ventricular hypertrophy, 52 weeks of sacubitril/valsartan produced a greater reduction in CMR-derived myocardial interstitial volume than valsartan alone [191]. This finding provides human imaging-based evidence that sacubitril/valsartan may reduce diffuse interstitial remodeling, although the relatively small sample size limits conclusions regarding clinical significance. In HFpEF, the larger PARAGON-HF trial demonstrated clinical outcome effects of sacubitril/valsartan but did not specifically assess myocardial fibrosis as a primary endpoint [192].

Sodium–glucose cotransporter 2 (SGLT2) inhibitors are now established therapies for heart failure and metabolic cardiovascular disease, but fibrosis-specific clinical evidence remains considerably more limited than their overall cardiovascular outcome data. In a biomarker analysis of the EMPEROR-Preserved and EMPEROR-Reduced trials, empagliflozin reduced circulating PICP at 12 and 52 weeks and PRO-C3 at 52 weeks, whereas several other markers of collagen synthesis and degradation remained unchanged [193]. These findings are consistent with an effect on collagen turnover but do not directly demonstrate regression of myocardial fibrosis. More recently, a small randomized study in patients with HFpEF and type 2 diabetes reported a greater reduction in CMR-derived extracellular volume with dapagliflozin than with placebo over 12 months [194]. Thus, imaging evidence supporting a potential antifibrotic effect of SGLT2 inhibition is beginning to emerge, but remains confined to selected patient populations and requires confirmation in larger studies.

Overall, established cardiovascular therapies differ substantially in both the type and strength of evidence supporting fibrosis-specific effects. ACEi/ARB therapy is supported by limited direct biopsy-based evidence, whereas evidence for MRAs remains predominantly biomarker-based. ARNIs have emerging CMR-based evidence of reduced diffuse interstitial remodeling, while the fibrosis-specific evidence for SGLT2 inhibitors is still largely based on circulating biomarkers, with imaging evidence currently limited to selected clinical populations. Despite these differences, these therapies act mainly by attenuating upstream neurohumoral, inflammatory, metabolic, and remodeling pathways rather than by directly targeting fibroblasts or the ECM. Their antifibrotic efficacy is therefore likely to vary with disease context and stage and may be greatest when fibrotic remodeling remains active and potentially reversible. Once extensive ECM accumulation, collagen cross-linking, and matrix stabilization have developed, conventional anti-remodeling therapy alone is unlikely to fully reverse established fibrosis (Table 3).

**Table 3:** Therapeutic strategies and translational evidence for cardiac fibrosis

Category	Strategy	Fibrosis-related evidence	Clinical development/status	References
Established therapy	ACEi/ARB	Direct evidence of myocardial fibrosis regression from small human endomyocardial biopsy studies;	Established clinical therapies; fibrosis-specific evidence derives mainly from small biopsy studies	[186, 187]
Established therapy	MRA	Circulating collagen biomarker evidence	Established therapy; fibrosis evidence mainly biomarker-based	[189, 190]
Established therapy	ARNI	CMR-based reduction in myocardial interstitial volume	Completed randomized phase 2 study	[191]
Established therapy	SGLT2 inhibitors	Collagen biomarkers; limited CMR evidence in selected diabetic HFpEF populations	Established heart failure therapy; direct fibrosis evidence remains limited	[193, 194]
Emerging therapy	Pirfenidone	CMR-derived ECV reduction in HFpEF	Completed randomized phase 2 trial	[195]
ECM-targeted	SNT-5382	Preclinical antifibrotic efficacy; human safety/target engagement	Completed phase 1; no patient efficacy established	[196]
ECM-targeted	Alagebrium	Randomized human trial evaluating AGE cross-link breaking; no demonstrated improvement in clinical/functional endpoints	Completed randomized clinical trial; negative efficacy findings	[197]
ECM-targeted	TG2 inhibition	Cellular and animal cardiac fibrosis evidence	Preclinical	[198]
Fibroblast-targeted	FAP-directed approaches	Reduced activated fibroblasts and fibrosis in mice	Preclinical	[199–201]
Fibroblast-targeted	CD248-targeted BBIR-T cells	Reduced post-MI fibrosis extension in mice	Preclinical	[202]

*Abbreviations*: *ACEi* angiotensin-converting enzyme inhibitor, *AGE* advanced glycation end product, *ARB* angiotensin receptor blocker, *ARNI* angiotensin receptor–neprilysin inhibitor, *BBIR-T* biotin-binding immune receptor T cell, *CMR* cardiovascular magnetic resonance, *ECM* extracellular matrix, *ECV* extracellular volume fraction, *FAP* fibroblast activation protein, *HFpEF* heart failure with preserved ejection fraction, *MI* myocardial infarction, *MRA* mineralocorticoid receptor antagonist, *SGLT2* sodium–glucose cotransporter 2, *TG2* transglutaminase 2

Pirfenidone is among the few emerging antifibrotic agents to have been evaluated using a direct imaging endpoint related to myocardial fibrosis. In the randomized, double-blind, phase 2 PIROUETTE trial, 94 patients with HFpEF and CMR-defined myocardial fibrosis were assigned to pirfenidone or placebo for 52 weeks. Pirfenidone produced a greater reduction in CMR-derived myocardial extracellular volume than placebo, with a between-group difference of −1.21% (95% CI, −2.12 to −0.31; *P* = 0.009) [195]. However, the trial was relatively small, and no significant differences were observed in several secondary functional outcomes, including diastolic function, 6-min walk distance, and health-status measures. These results therefore provide phase 2 human imaging evidence that pirfenidone can reduce myocardial interstitial fibrosis, but whether this translates into meaningful clinical benefit remains to be established in larger outcome trials.

Targeting ECM cross-linking represents another emerging antifibrotic approach. SNT-5382 is a small-molecule inhibitor of lysyl oxidase-like 2 (LOXL2), an enzyme involved in collagen cross-linking and matrix stabilization. In a mouse model of MI, SNT-5382 reduced cardiac fibrosis and improved cardiac function [196]. The compound subsequently entered phase 1 clinical evaluation, in which single- and multiple-ascending-dose studies in healthy volunteers demonstrated acceptable tolerability, favorable pharmacokinetic characteristics, and sustained LOXL2 target engagement. Importantly, these phase 1 studies were designed to assess safety, pharmacokinetics, and target engagement rather than antifibrotic efficacy in patients with heart failure. Thus, although SNT-5382 has demonstrated preclinical antifibrotic activity and early clinical feasibility, its therapeutic efficacy against cardiac fibrosis in patients remains to be determined.

Alagebrium, an advanced glycation end-product (AGE) cross-link breaker, has also been investigated as a means of modifying ECM cross-linking and myocardial stiffness. In the randomized, double-blind, placebo-controlled BENEFICIAL trial, 102 patients with chronic heart failure and left ventricular systolic dysfunction received alagebrium or placebo for 36 weeks. Alagebrium did not improve the primary endpoint of exercise capacity, and no significant benefits were observed across several secondary measures of cardiac function [197]. Thus, although disruption of AGE-mediated matrix cross-linking remains mechanistically relevant, this trial did not demonstrate therapeutic efficacy of alagebrium in chronic systolic heart failure.

Transglutaminase 2 (TG2) represents another potential ECM-related therapeutic target because of its involvement in protein cross-linking, matrix deposition, and TGF-β1 signaling. In vitro, the selective TG2 inhibitor 1–155 attenuated TGF-β1-induced fibroblast-to-myofibroblast transition, endothelial-to-mesenchymal transition, and matrix deposition. In vivo, TG2 inhibition reduced collagen deposition and TG2-mediated protein cross-linking in mouse models of both Ang II-induced interstitial fibrosis and MI [198]. These findings support TG2 inhibition as a potential preclinical antifibrotic strategy, although its efficacy and safety in patients with cardiac fibrosis remain to be established.

#### Targeted and engineered intervention strategies

Fibroblast-targeted immunotherapy has emerged as an experimental approach for selectively eliminating activated fibroblasts. Fibroblast activation protein (FAP), which is enriched in activated cardiac fibroblasts during pathological remodeling, has been investigated as a therapeutic target. In mouse models of cardiac injury, adoptive transfer of FAP-directed chimeric antigen receptor (CAR) T cells reduced cardiac fibrosis and improved cardiac function, providing proof of concept that activated fibroblasts can be therapeutically targeted [199]. A subsequent study developed a transient in vivo CAR-T strategy using modified mRNA encapsulated in T-cell-targeted lipid nanoparticles. This approach generated transient FAP-directed CAR T cells in vivo, reduced cardiac fibrosis, and improved cardiac function in a mouse model of cardiac injury [200]. Together, these studies demonstrate the feasibility of FAP-directed cellular immunotherapy in preclinical models of cardiac fibrosis; however, its safety, target specificity, and therapeutic efficacy in humans remain unknown. Vaccination against FAP represents an alternative strategy for targeting activated fibroblasts. In a murine model of chronic cardiac stress induced by continuous administration of angiotensin II and phenylephrine, a FAP peptide vaccine combined with a CpG K3 adjuvant increased anti-FAP antibody titers, reduced the accumulation of FAP-positive myofibroblasts, and decreased cardiac fibrosis [201]. No systemic or organ-specific inflammation attributable to antibody-dependent cytotoxicity was detected, and no adverse effects were observed in additional models of MI or skin injury. These findings provide preclinical proof of concept for FAP-directed vaccination, although its long-term safety, specificity, and therapeutic efficacy in humans remain to be determined.

A more selective fibroblast-targeting strategy has recently been developed against CD248, a surface marker enriched in a late-activated fibroblast subpopulation after MI. Using single-cell RNA sequencing, Chen et al. identified CD248 as a characteristic antigen of these late-activated fibroblasts and developed a biotin-binding immune receptor T-cell (BBIR-T) approach to target them after scar maturation. In a mouse model of MI, CD248-directed BBIR-T treatment initiated 14 days after infarction reduced fibrotic expansion in the peri-infarct region, attenuated adverse ventricular remodeling, and delayed deterioration of cardiac function [202]. These findings suggest that temporally and phenotypically selective targeting of pathological fibroblast subpopulations may suppress excessive fibrosis while preserving the reparative scar response required during the early phase of myocardial healing. However, this approach remains at the preclinical stage, and its safety, specificity, and translational feasibility in humans require further investigation.

#### Targeting SIRT3: therapeutic prospects and translational challenges

Current strategies for therapeutic modulation of SIRT3 include pharmacological activators or modulators, NAD^+^-related metabolic interventions, and targeted gene delivery. The synthetic small molecule 2-APQC has been identified as a SIRT3 activator and was shown to attenuate isoproterenol-induced myocardial hypertrophy and fibrosis in rodent models [203]. These protective effects were largely abolished in SIRT3-deficient mice, supporting a SIRT3-dependent mechanism in this experimental setting. Honokiol [204, 205] and colchicine [206] have also been reported to modulate SIRT3-associated pathways in experimental cardiac models. Across these studies, SIRT3 modulation was associated with improved mitochondrial function and redox homeostasis and attenuation of pathological cardiac remodeling, although the underlying mechanisms and disease models differed among the agents (Table 4).

**Table 4:** SIRT3-modulating strategies relevant to cardiac fibrosis

Category	Strategy	Evidence	Translational status	References
Small-molecule activators	2-APQC	Direct SIRT3 activation; attenuated ISO-induced cardiac hypertrophy and fibrosis in a SIRT3-dependent manner	Preclinical	[203]
Small-molecule activators	Honokiol	Enhanced SIRT3 activity and mitochondrial protein deacetylation; attenuated pathological cardiac remodeling in experimental models	Preclinical	[204, 205]
Pharmacological modulation	Colchicine	Reduced fibrosis and oxidative stress in pressure overload-induced remodeling in association with SIRT3 activation	Preclinical SIRT3-related evidence	[206]
Metabolic interventions	Nicotinamide riboside (NR)	Increased NAD⁺ availability in HFrEF	Early human evidence; no fibrosis-specific efficacy	[207]
Metabolic intervention	Exogenous NADPH	Enhanced SIRT3-mediated mitochondrial metabolism	Preclinical	[208]
Targeted delivery	UTMD-mediated SIRT3 delivery	Increased cardiac SIRT3 expression and reduced remodeling	Large-animal preclinical	[181]

*Abbreviations*: *HFrEF* heart failure with reduced ejection fraction, *ISO* isoproterenol, *NAD⁺* nicotinamide adenine dinucleotide, *NADPH* reduced nicotinamide adenine dinucleotide phosphate, *NR* nicotinamide riboside, *SIRT3* sirtuin 3, *UTMD* ultrasound-targeted microbubble destruction

Because SIRT3 is an NAD^+^-dependent deacetylase, strategies that increase NAD^+^ availability have also been explored as indirect means of supporting SIRT3-mediated mitochondrial regulation [207, 209].

In a small clinical study of patients with stable heart failure with reduced ejection fraction (HFrEF), nicotinamide riboside (NR) was well tolerated and approximately doubled whole-blood NAD^+^ levels [207]. However, the study did not assess myocardial fibrosis or establish that any clinical effects were mediated through SIRT3. By contrast, evidence for exogenous NADPH remains predominantly preclinical. In experimental models of pathological cardiac hypertrophy and heart failure, NADPH increased SIRT3 expression and activity, enhanced mitochondrial protein deacetylation, and improved cardiac energetics and function [208]. Targeted gene delivery represents another experimental strategy. Ultrasound-targeted microbubble destruction (UTMD)-mediated SIRT3 delivery increased cardiac SIRT3 expression and attenuated adverse remodeling in a porcine model of pathological cardiac hypertrophy [181]. Thus, although metabolic and gene-delivery approaches provide alternative routes for modulating SIRT3-related pathways, their fibrosis-specific clinical efficacy has not yet been established.

Taken together, SIRT3-directed interventions should not be regarded as universally applicable or stand-alone antifibrotic therapies. Their therapeutic relevance is likely to depend on the underlying disease context, the dominant cellular and metabolic drivers of remodeling, and the stage of fibrotic progression. SIRT3 modulation may be particularly relevant when mitochondrial dysfunction, oxidative stress, and metabolic disturbances remain active contributors to remodeling, whereas its effects may be more limited once extensive ECM deposition, collagen cross-linking, and matrix stabilization have become established. Future studies should therefore define the disease settings, cellular targets, and therapeutic windows in which SIRT3 modulation is most likely to confer meaningful benefit.

## Conclusion and outlook

Cardiac fibrosis is not simply a static endpoint characterized by excessive ECM deposition; rather, it is a dynamic remodeling response to diverse pathological stimuli [1, 210]. In some settings, fibrotic remodeling is essential for maintaining tissue integrity and facilitating repair. Under persistent pathological stress, however, this response can become maladaptive and contribute to increased myocardial stiffness, electrical conduction abnormalities, and progression of heart failure. The central challenge is therefore not merely to identify profibrotic pathways, but to determine when fibrosis becomes pathological, in which disease contexts it is most consequential, and at what stage therapeutic intervention is likely to be most effective [211].

Advances in lineage tracing, single-cell transcriptomics, and epigenetic profiling have substantially refined our understanding of fibroblast state transitions. Cardiac fibrosis is now viewed less as a morphological process defined solely by collagen accumulation and more as a dynamic pathological program shaped by cellular heterogeneity, microenvironmental remodeling, and temporally regulated cell-state transitions. Several important limitations nevertheless remain. First, mechanistic findings derived from different experimental models and disease contexts are highly context dependent and cannot be readily generalized across pathological settings. Second, many profibrotic mediators may exert different, and in some cases opposing, biological effects during acute tissue repair and chronic remodeling. Third, longitudinal relationships linking experimental models, human myocardial tissue, and clinically relevant phenotypes remain incompletely defined.

SIRT3 is of particular mechanistic interest in cardiac fibrosis because it integrates mitochondrial homeostasis, oxidative stress, metabolic adaptation, and profibrotic signaling. Rather than constituting an isolated antifibrotic pathway, SIRT3 may provide a mechanistic framework for understanding how mitochondrial and metabolic abnormalities interact with fibroblast activation and matrix remodeling. Its effects, however, appear to vary according to disease context, cell type, and stage of remodeling, and SIRT3 should therefore not be considered a universally applicable therapeutic target. Future studies should identify the pathological settings and therapeutic windows in which mitochondrial and metabolic vulnerability remains an active driver of fibrosis and determine whether these features can be incorporated into more precise, mechanism-based antifibrotic strategies.

## Data Availability

Not applicable.
